# Pulmonary and clinical outcomes of patients with severe rigid scoliosis and type I respiratory failure treated with halo-pelvic distraction

**DOI:** 10.1186/s13018-023-04212-7

**Published:** 2023-09-21

**Authors:** Deng Zhao, Fei Wang, Zhengjun Hu, Rui Zhong, Yijian Liang

**Affiliations:** Department of Orthopaedics, The Third People’s Hospital of Chengdu/The Affiliated Hospital of Southwest Jiaotong University, Chengdu, China

**Keywords:** Severe rigid spinal deformity, Halo‐pelvic distraction, Pulmonary function, Type I respiratory failure

## Abstract

**Background:**

The severe rigid scoliosis patients with type I respiratory failure could not tolerate complicated corrective surgery. Preoperative halo-pelvic distraction (HPD) is used to reduce the curve magnitude and improve the pulmonary function before surgery. The present study aimed to retrospectively analyze the pulmonary and clinical outcomes of preoperative HPD in severe rigid spinal deformity with type I respiratory failure.

**Methods:**

Eighteen cases of severe rigid scoliosis and type I respiratory failure treated with preoperative HPD and corrective surgery for spinal deformity between 2016 and 2018 were retrospectively reviewed. Patient demographics, major coronal curve and kyphosis, correction rates, heights, pulmonary function, distraction time, and postoperative neurological complications were recorded for all cases.

**Results:**

The averaged duration of distraction was 9.1 ± 2.3 months. The coronal curve was corrected from 168° ± 14° to 58° ± 11° at the end of HPD. The kyphosis curve reduced from 151° ± 29° to 65° ± 10°. Meanwhile, the mean stand body height increased by 23.9 ± 5.3 cm. Significantly increased mean FVC (1.52 ± 0.43 L vs. 0.95 ± 0.44 L) and improved percent-predicted values for FVC (37 ± 10% vs. 23 ± 9%) were observed after HPD. The pressure of oxygen (PaO2) increased from 54.5 ± 2.0 to 84.8 ± 4.7 mmHg. Scoliosis and kyphosis curve, respectively, averaged 48 ± 8°and 30 ± 14° after final fusion and instrumentation, with a mean correction of 71% and 80%, respectively. No severe complication occurred during the distraction.

**Conclusions:**

HPD may be useful for severe rigid scoliosis patients with type I respiratory failure. Pulmonary functions in patients with severe rigid scoliosis can be significantly improved by HPD. They are then better able to tolerate complicated corrective surgery.

## Background

Correction of severe and rigid scoliosis remains a surgical challenge, especially in patients with severe pulmonary impairment, despite decades of improvement in surgical techniques and instrumentation [1, 2]. Factors contributing to the pre-existing severe pulmonary dysfunction include restrictive lung disease due to scoliosis, deformed rib cage, reduced chest wall compliance, impairment of chest mechanics and obstructive lung disease due to large airway compression [3–5]. The high morbidity of perioperative respiratory failure makes it impossible to provide effective correction [6].

The aim of surgery in these patients is to correct the curve and to restore trunk balance while improving the patient’s quality of life and pulmonary function [7, 8]. Spinal osteotomy under intraoperative monitoring could be effective for severe and rigid scoliosis. But the surgery procedures for severe rigid scoliosis have been recognized as substantially risky and high demanding, requiring complex osteotomy and prolonged high-intensity or even multiple surgeries, and is associated with serious complications such as pulmonary complications, massive blood loss and neurological injury [9, 10]. Patients with severe scoliosis and severe pulmonary dysfunction could not tolerate such complicated corrective surgery. Therefore, preoperative distraction such as halo-pelvic distraction (HPD) was recommended to increase the safety and correction rate of staged surgery for patients with severe rigid scoliosis and severe pulmonary dysfunction.

Preoperative halo-pelvic distraction (HPD) was developed to achieve a gradual correction since first reported by O’Brien et al. [11]. It has been reported as a safe method for staged correction of severe and rigid scoliosis. Excellent clinical results have been reported, although the procedure has not been popular recently because of the significant shortcoming such as long wearing time of the cumbersome apparatus, restraining patients much and challenges in appearance and daily life [12–15]. Despite these shortcomings, HPD provides a safe method for treating cases of scoliosis associated with severe respiratory dysfunction. The deformed rib cage may improve following the gradual correction of the scoliosis. Additional respiratory training could be performed to coordinate the functions of respiratory muscles to improve respiratory function while the device allows patients’ mobility.

In this report, we presented a series of patients with severe and rigid scoliosis and type I respiratory failure (defined as PO_2_ < 60 mmHg and normal PCO_2_) who underwent staged surgical management. It includes HPD for gradual correction of scoliosis and improvement of respiratory function, and spinal osteotomy and fixation for final correction. For patients with severe scoliosis and respiratory dysfunction bring great challenges for surgeons, anesthesiologist. It can be a safe way using HPD and spinal surgery to save their life, correct the deformity and improve their quality of life.

## Methods

### Patient recruitment

A series of 18 adult scoliosis patients with pre-existing respiratory failure (8 males and 10 females, of which one case presented chronic cardiopulmonary failure on admission) undergoing preoperative HPD combined with respiratory training were retrospectively reviewed. The etiological diagnoses of the cases were idiopathic scoliosis (IS, 14 cases), congenital scoliosis (CS, 3 cases), Marfan syndrome (MF, 1 case). Inclusion criteria consisted as follows: (1) ≥ 18 years; (2) major thoracic curve (apical vertebra located in T1-T11) more than 150°; (3) preoperative respiratory dysfunction defining as forced vital capacity predicted (FVC%) < 35%; (4) type I respiratory failure with pressure of oxygen (PaO_2_) < 60 mmHg and pressure of carbon dioxide (PCO_2_) < 50 mmHg in arterial blood gas analysis [1, 16]. All the patients underwent respiratory training, such as deep respiration and balloon exercise, physical exercises, and nutritional support. All the patients underwent staged treatment, first HPD until the pulmonary function improved enough, and second spinal corrective surgery. The minimum follow-up was 36 months. The study was approved by the ethics committee of the Third People's Hospital of Chengdu.

### Radiographic analysis

The major scoliosis and kyphosis was extracted from biplanar, full-standing, standard radiographs preoperatively, post-operatively as well as radiographs performed with patients in HPD. Preoperative MRI was assessed for intra-spinal anomalies. Three surgeons independently measured the major curve of scoliosis and kyphosis on pre-distraction, post-distraction and post-operative long-cassette standing radiographs. Repeated spinal plain films were performed every 2 months to observe the changes of the main curve of scoliosis and kyphosis.

### Pulmonary function test

Pulmonary function was measured using a pulmonary function test instrument. The pulmonary function value reported was forced vital capacity (FVC) and the percentage of the predicted value (FVC%). Pre-distraction pulmonary function tests (PFT) and repeated PFTs till the completion of distraction were performed. The highest values of PFTs repeated three times at each evaluation were used. Arm span was used instead of height to calculate age-based predicted normal values because of the height was changing resulted by gradual correction of spinal deformity [17]. According to the American Thoracic Society’s guidelines for the severity of pulmonary impairment [18], ‘no’ pulmonary impairment was considered when the FVC was > 80% of the predicted value, ‘mild’ when the FVC was ≤ 80% but > 65%, ‘moderate’ when the FVC was ≤ 65% but > 50%, and ‘severe’ when the FVC was ≤ 50%. The parameters of PFTs included forced vital capacity (FVC), FVC % predicted. Arterial blood gas analysis was used to define the respiratory failure. The respiratory failure was classified in 2 types. Severe arterial hypoxemia that is refractory to supplemental O_2_ (PaO_2_ < 60 mm Hg) with normal or low arterial carbon dioxide tension (PaCO_2_) is defined as type I respiratory failure also called hypoxic respiratory failure. Type II respiratory failure also called hypoxic respiratory failure is defined as a PCO_2_ > 50 mmHg.

### Halo-pelvic distraction

**Fig. 1:**
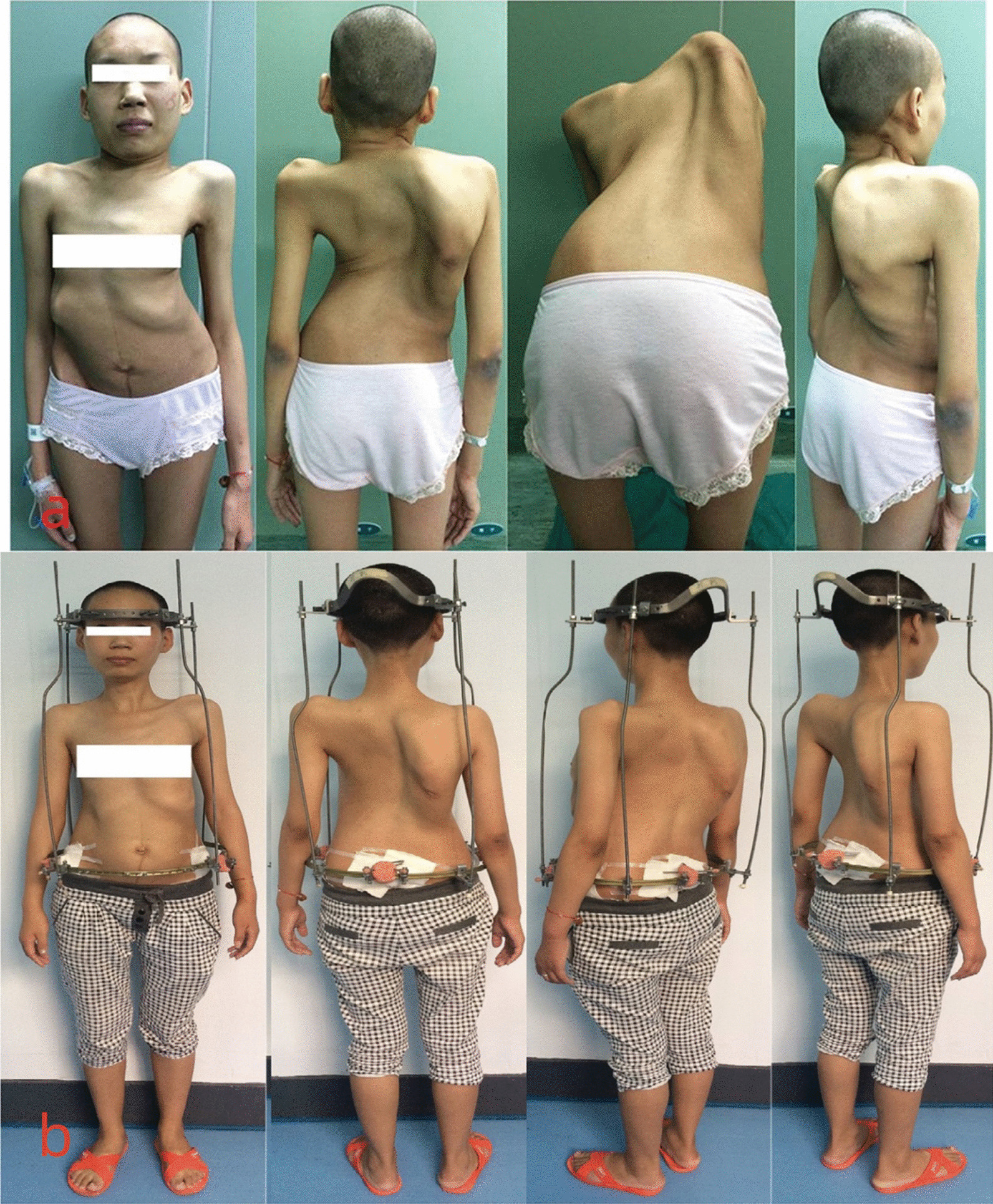
A 36 years female patient with severe rigid scoliosis and respiratory failure. **a** The appearance of the patient on admission; **b** the appearance of the patient with halo-pelvic distraction

**Fig. 2:**
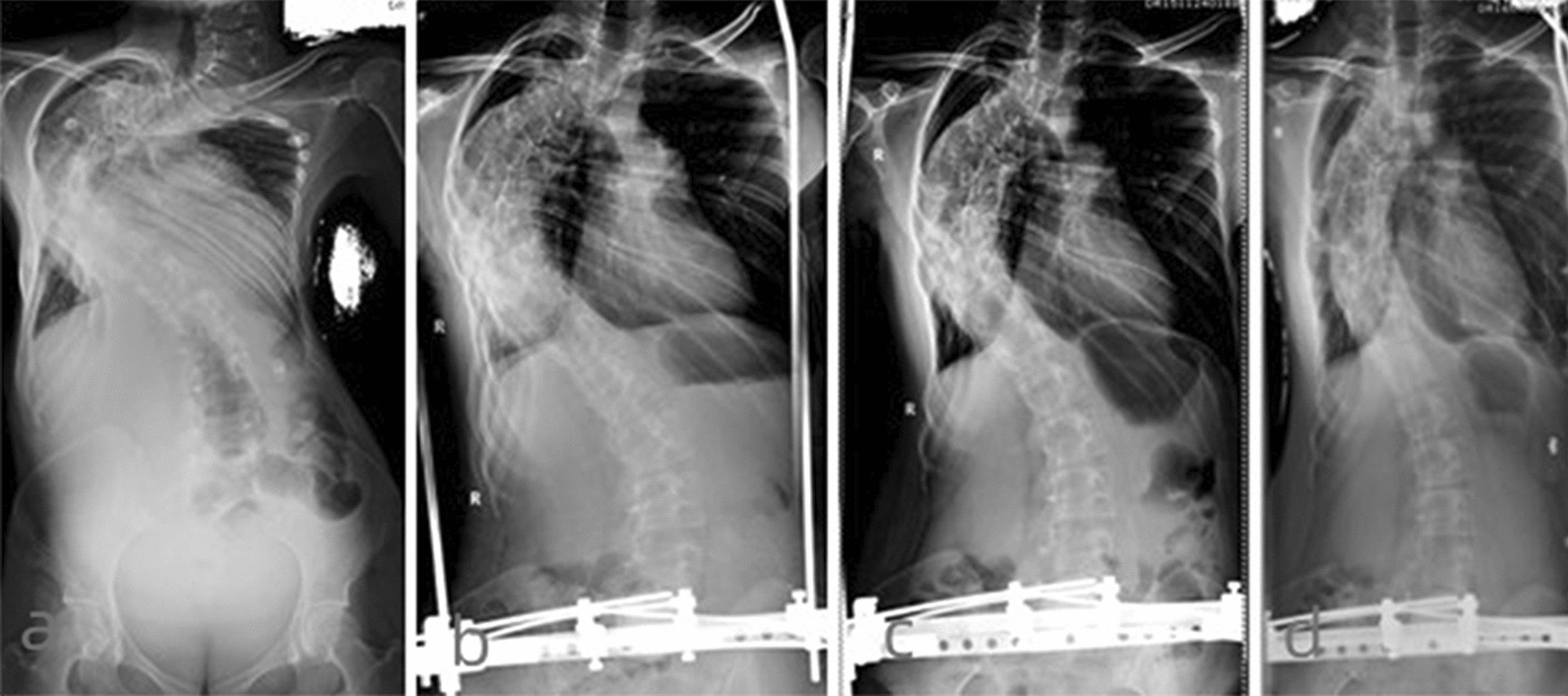
The anterior–posterior film of the 36 years female patient with severe rigid scoliosis and respiratory failure. **a** major curve was 152° on admission; **b** major curve reduced to 104° after 3 months distraction; **c** major curve reduced to 83° after 6 months distraction; **d** major curve reduced to 64° after 9 months distraction;

The device features halo and pelvic rings fastened to the cranial and iliac bones, respectively, and four correcting rods that align the two rings to allow spinal distraction. General anesthesia is essential during fitting. The halo frame was placed immediately above the right skull equator and fastened using ten pins tightened to 6–8 in of torque. Two pelvic ring pins were placed on the iliac crest and the posterior superior iliac. After the patient could adapt to it and develop confidence with daily life, the whole halo pelvic apparatus was fitted (Fig. 1). The distraction began. The bars were distracted approximately 5 mm each week (Fig. 2). We carefully monitored whether the neurological deficit occurred. The distraction would be temporarily static as if distraction related complains occurred such as neck pain, numbness. Respiratory training was performed including deep respiration, abdominal respiration, lip-constriction respiration, balloon exercise during the distraction. Appropriate physical exercise to improve cardiopulmonary reserve and nutritional support to restore optimal nutrition status and health were also performed. Surgery may be considered if the PFT results reached plateau and they may tolerate complicated surgery and if the curve improvement was judged to be maximized. If the pulmonary function did not improve satisfactory, a posterior release surgery would be considered.

### Statistics

All data were analyzed using statistical software (SPSS 20.0, SPSS Inc, Chicago IL). Statistical data were presented as the mean ± standard deviation. The changes in PFTs and radiographic parameters of pre- and post-distraction were compared using paired Student’s* t* test. Statistical significance was defined as *P* < 0.05.

## Results

The mean age of 18 patients ( 8 male and 10 female) prior to HPD was 25.3 ± 3.6 years (range, 19–30 years). Follow-up ranged from 36 to 60 months (mean, 46.8 ± 8.8 months). The initial major coronal curve averaged 168° ± 14° (150° to 180°) and kyphosis angle averaged 151° ± 29° (90° to 180°) as shown in Table 1. The averaged duration of distraction was 9.1 ± 2.3 months (6 to 12 months). The bars were distracted approximately 5 mm each week. At the end of HPD, mean major coronal curve significantly decreased to 58° ± 11° (*P* < 0.001) and kyphosis angle averaged to 65° ± 10° (*P* < 0.001). Meanwhile, the stand body height increased by 23.9 ± 5.3 cm (from 136.1 ± 10.5 cm to 160.0 ± 11.4 cm, *P* < 0.001).

**Table 1:** Radiographic parameters description of the severe scoliotic patients

	Pre-distraction	Post-distraction	Post-operation	Correction rate(%)
Major coronal curve (°)	168° ± 14°	58° ± 11°	48° ± 8°	71
Kyphosis (°)	151° ± 29°	65° ± 10°	31° ± 14°	80
Height (cm)	136.1 ± 10.5		160.0 ± 11.4	–

Regarding results of PFT, significantly increased mean FVC (1.52 ± 0.43 L vs. 0.95 ± 0.44 L, *P* < 0.05) and improved percent-predicted values for FVC (37 ± 11% vs. 23 ± 9%, *P* < 0.05) were observed after HPD. The pressure of oxygen (PaO2) increased from 54.5 ± 2.0 mmHg to 84.8 ± 4.7 mmHg. All of the patients were with severe pulmonary impairment on admission according to the American Thoracic Society’s guidelines for the severity of pulmonary impairment (Table 2).

**Table 2:** FVC and FVC% of PFT description of the patients

	Pre-distraction	Post-distraction	Improvement rate (%)
FVC (L)	0.95 ± 0.44	1.52 ± 0.43	60
FVC%	23 ± 9	37 ± 11	66
PO_2_(mmHg)	54.5 ± 2.0	84.8 ± 4.7	56
PCO_2_(mmHg)	45.2 ± 2.3	45.3 ± 1.7	–

Two patients suffered pelvic pin site infections which requiring antibiotics. One patient underwent pelvic pin exchange, because one pin dislodged due to osteoporosis and the significant force of distraction. No other halo-pelvic distraction relative complications were recorded. No neurological complication was observed in all 18 patients during HPD.

**Fig. 3:**
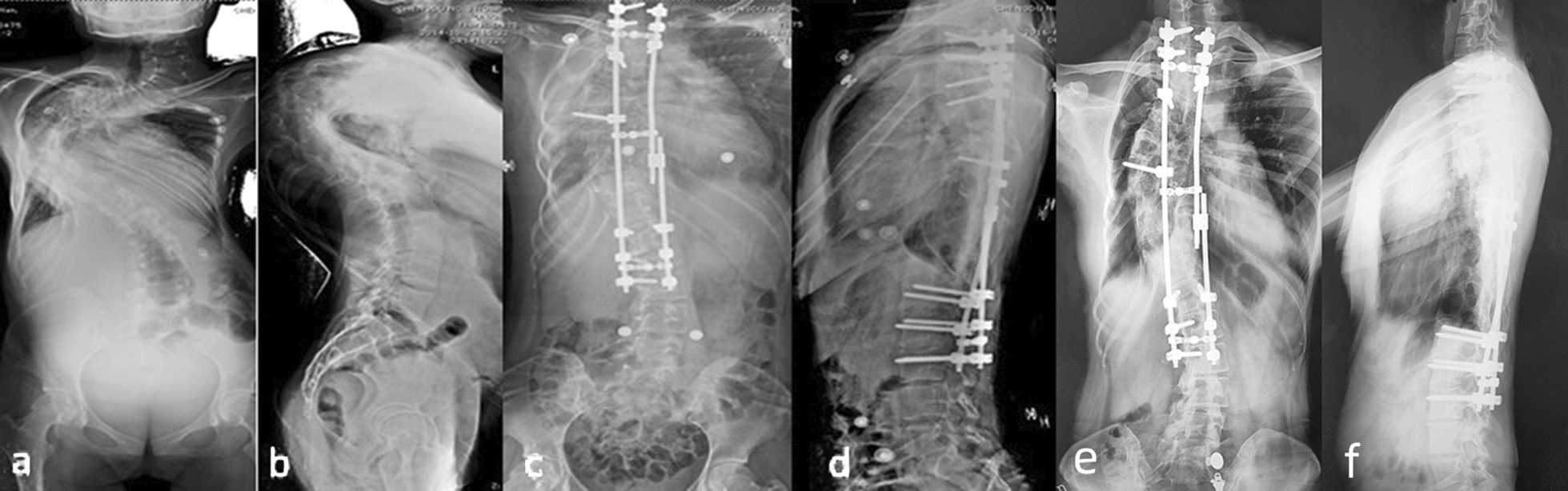
Preoperative application of HPD in the 36-year-old female with a severe rigid scoliosis and respiratory failure. **a**, **b**, the initial whole standing spine films, with a major curve of 152° and kyphosis 149°; **c**, **d**, post-operative whole standing spine film after 9 months HPD, with a major curve of 45°and kyphosis 30°, which was showed in Fig. 2; **e**, **f**, the whole standing spine film at approximately 5-year follow-up, with a major curve of 47° and kyphosis 30°

**Fig. 4:**
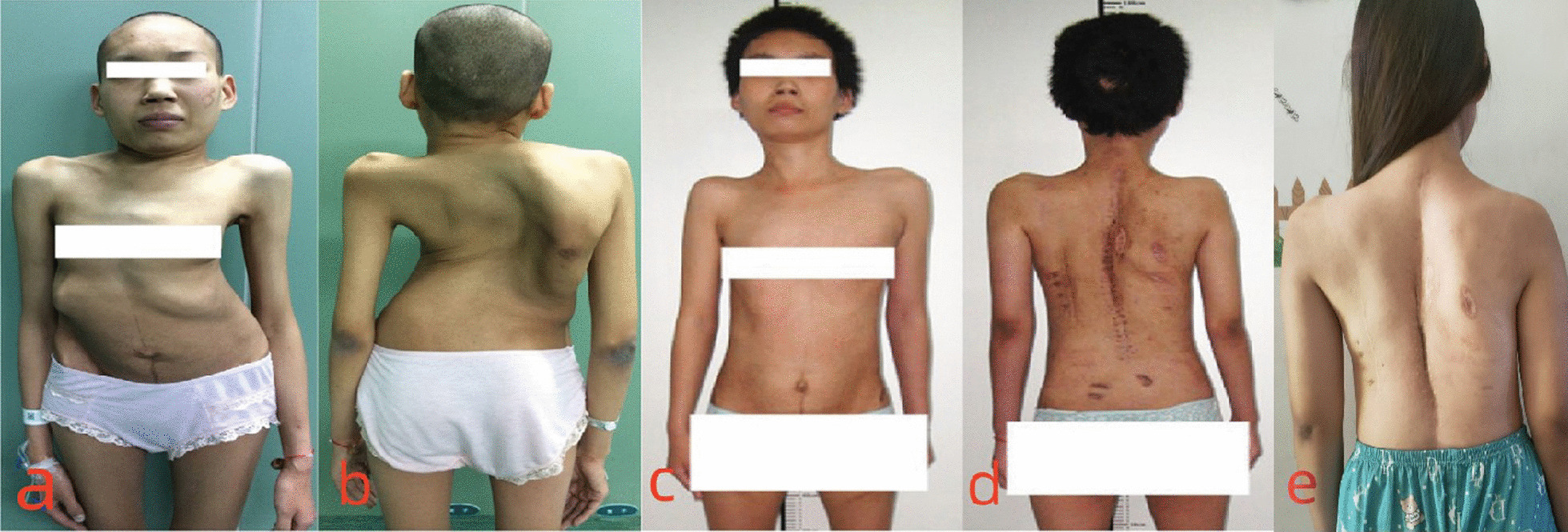
The 36 years female patient with severe rigid scoliosis and respiratory failure. **a**, **b** The appearance of the patient on admission; **c**, **d** the appearance of the patient after final corrective surgery; **e** the appearance of the patient at approximately 5-year follow-up

Three patients underwent posterior releases including soft-tissue and facetectomies releases during distraction. One-stage posterior-only correction was performed for all patients when the distraction was enough. The posterior instrumentation was all pedicle screw system without hybrid hooks. At the final corrective surgery, vertebral column resection (VCR) was performed in 4 patients, the remaining 14 cases underwent only facetectomies. The major coronal curve and kyphosis averaged 48 ± 8 degrees and 31 ± 14 degrees after corrective surgery with mean correction of 71% and 80%, respectively (Fig. 3). The clinical outcome was satisfied (Fig. 4). SEP/MEP was used to monitor if neurological complication occurred during the surgeries, and the results of SEP/MEP were negative. No post-operative respiratory complication was recorded.

## Discussion

Surgical treatment on severe scoliosis complicated with respiratory impairment remains challenges for resulting in significant increase in morbidity and mortality compared to moderate spinal deformity. Weinstein et al. [19] reported that clinically relevant pulmonary impairment would occur once curves progress beyond 100°, with strong correlations between curve severity and results of PFT. Several method for gradual correction of scoliosis curve in severe scoliosis have been developed, such as halo-pelvic distraction (HPD), halo-gravity traction (HGT), halo-femoral traction (HFT) [11, 20, 21]. Effective clinical outcomes have been reported for HGT [17, 22], while HPD reported by some case reports is not so popular for its shortcomings [12–15]. The primary goal of the method is to improve pulmonary function, avoid major neurological risk and to obtain correction of severe scoliosis.

Severe scoliosis and kyphosis affect the thoracic cage, disturbing skeletal, muscular and diaphragmatic function and reducing respiratory system compliance [23]. Respiratory function, rather than curve size, is the predominant problem, which has a bearing on physical function and mortality. Early surgery is recommended and is said to avoid progressing pulmonary impairment, and increase in life expectancy [1, 24, 25]. However, the multifactorial genesis of pulmonary impairment is beyond the effect of spinal surgery which is successful in curve correction [25].

Significant improvement in results of FVC of PFTs was observed after combined HPD and additional respiratory training in our report. The average improvement rate of FVC and FVC% were, respectively, 60% and 66%. For severe scoliosis patients with extreme pulmonary impairment, to improve their pulmonary function and quality of life should be the first goal while severe respiratory complication easily following the spinal surgery. Kanagaraju et al. [13]. reported a case of severe and rigid congenital thoracolumbar lordoscoliosis with type 2 respiratory failure whose lung condition did not allow for any major procedure underwent a staged procedure including controlled axial distraction, posterior release, halo-pelvic distraction and osteotomy, and excellent outcome was achieved. In this report a similar patient was included. The patient with chronic cardiopulmonary failure on admission who presented worsening dyspnea and pitting edema of the lower extremities, to improvement the cardiopulmonary function to survive was the first goal for treatment. Maybe assisted ventilation with other conservative treatment was a choice, but she might not be able to survive without these. Surgical treatment might be contraindicated, while the PO2 was 50.6 with oxygen therapy and BNP was 2712 ng/ml. During the preoperative distraction, the cardiopulmonary function improved.

Preoperative distraction supplies a safe method for cases of severe scoliosis with severe pulmonary impairment to improve their lung condition and reduce the high rate of intraoperative and postoperative morbidity and mortality. Koller et al. [22] has reported a significant improvement in pulmonary function after HGT, and the average change of FVC% was 7 ± 8% (n = 24). In the previous studies, pulmonary function was reported to benefit from preoperative HGT with mean improvement of FVC% ranging from 10 to 14% [6, 26, 27]. Bao et al. [17] reported a series cases indicated that combined HGT and assisted ventilation were efficient not only in pediatrics but also in adults. To our knowledge, this is the first report on staged treatment of scoliosis patients with the major curve exceeded 150 degree and type I respiratory failure using HPD, assisted respiratory training and spinal osteotomy, and the result was excellent. In this reported the FVC% improved to 37% from 23%. Both restrictive and obstructive lung disease were observed in these severe scoliosis cases. Obstructive lung disease may be due to large airway compression, which could be improved as the mild improvement of severe deformity after HPD. Meanwhile, enlarging of thoracic cavity and increased contraction ability of respiratory muscle after HPD and assisted respiratory training improved the restrictive lung disease blamed for decreased lung volume [23]. After HPD, the PO2 was improved to normal in all patients.

Patients with severe rigid spinal deformity present a formidable challenge to the spine surgeon to achieve adequate correction. Several different surgical procedures were available for treating rigid spinal deformities, including Smith-Petersen osteotomy (SPO), pedicle subtraction osteotomy (PSO), vertebral column resection (VCR), and even higher level of spinal osteotomy [9, 28]. These techniques take a one-stage correction for spine deformity, and undoubtedly increase surgical-related complications in severe and rigid scoliosis cases. Patients with severe and rigid scoliosis may easily suffer neurologic deteriorations in these one-stage procedures while the spinal cord has poor tolerance for tension and/or shrinking. Preoperative distraction provides a slow and safe correction of deformity [12]. A posterior-only approach with segmental pedicle screw instrumentations could not usually achieve a correction of > 50% in curves > 90° [22]. Though the number of reports of correction rates of ~ 50–65% in rigid curves is growing with a VCR, temporary neurologic deficits have been seen in up to 30% of patients [29–31]. An effectively preoperative distraction in patients with severe scoliosis may improve surgical safety for both correcting deformity gradually, and helping to decrease the risk of neurological or respiratory complications [32]. Bao et al. [17] reported none of 21 adult patients with severe scoliosis treated by HGT and spinal osteotomy suffered neurological or respiratory complications.

In our report, halo-pelvic distraction was well tolerated with no severe complications. The correction rate of distraction was approximately 65% and 57% of coronal major curve and kyphosis, respectively. The reports about HGT and HFT demonstrated the correction rate of traction was about no more than 50% [17, 20, 22, 27, 33]. Despite numerous evidences supporting HGT, several arguments on the benefits of HGT on severe scoliosis were reported that no improvement was observed with HGT for surgical outcomes [34, 35]. Koller et al. [22] reported that preoperative HGT should not be expected to significantly improve severe curves without a prior anterior and/or posterior release. But preoperative distraction in patients with severe scoliosis benefit not only improving spinal deformity gradually, but also decreasing the risk of neurological and respiratory complication. In this study, the HPD improved the major curve much more than the HGT or HFT in previous reports. The PFTs improved also better than patients with HGT or HFT reported. The advantage of HPD over HGT/HFT on improving spinal deformity and pulmonary function might be related with following reasons: (1) the corrective force is much stronger than HGT/HFT, the persistent and gradual distraction is powerful enough to improve the severe and rigid scoliosis; (2) the distraction persists 24-h; (3) patients can take assist respiratory training including deep respiration and balloon exercise, physical exercises including climb stairs and chest-expanding exercises, and nutritional support with the halo pelvic apparatus freely.

## Data Availability

Data and materials contributing to this article may be provided by sending an e-mail to the first author.
